# Co-Delivery of Loxoprofen and Tofacitinib by Photothermal Microneedles for Rheumatoid Arthritis Treatment

**DOI:** 10.3390/pharmaceutics15051500

**Published:** 2023-05-15

**Authors:** Yi Lu, Ting Xiao, Rongrong Lai, Ziyi Liu, Weixuan Luo, Yixuan Wang, Shijia Fu, Guihong Chai, Jinjing Jia, Yuehong Xu

**Affiliations:** 1 School of Pharmaceutical Sciences, Sun Yat-sen University, Guangzhou 510006, China; 2 State Key Laboratory of Dampness Syndrome of Chinese Medicine, The Second Affiliated Hospital of Guangzhou University of Chinese Medicine, Guangzhou 510120, China; 3 Department of Dermatology, The Second Affiliated Hospital of Guangzhou University of Chinese Medicine, Guangzhou 510120, China; 4 Guangdong-Hong Kong-Macau Joint Lab on Chinese Medicine and Immune Disease Research, Guangzhou University of Chinese Medicine, Guangzhou 510120, China

**Keywords:** loxoprofen, tofacitinib, microneedle, photothermal therapy, rheumatoid arthritis

## Abstract

Rheumatoid arthritis (RA) is an autoimmune disease of synovial inflammation that affects populations worldwide. Transdermal drug delivery systems for treating RA have increased but remain challenging. We fabricated a dissolving microneedle (MN) system with photothermal (PT) polydopamine (PDA) to co-load the non-steroidal anti-inflammatory drug loxoprofen (Lox) and the Janus kinase inhibitor tofacitinib (Tof), with the aim of co-delivering Lox and Tof directly to the articular cavity, aided by the combination of MN and PT. In vitro and in vivo permeation studies showed that the PT MN significantly promoted drug permeation and retention in the skin. An in vivo visualization of the drug distribution in the articular cavity showed that the PT MN significantly promoted drug retention in the articular cavity. Importantly, compared to the intra-articular injection of Lox and Tof, the application of the PT MN to a carrageenan/kaolin-induced arthritis rat model exhibited superior performance in reducing joint swelling, muscle atrophy, and cartilage destruction. Furthermore, the PT MN downregulated the mRNA expression levels of proinflammatory cytokines, including TNF-*α*, IL-1*β*, iNOS, JAK2, JAK3, and STAT3. The results show that the PT MN transdermal co-delivery of Lox and Tof is a new synergetic therapy with high compliance and good therapeutic efficacy for RA.

## 1. Introduction

With a global incidence of 1%, rheumatoid arthritis (RA) is a serious autoimmune disease of the joints [1]. The clinical symptoms of RA are mainly manifested as synovial hyperplasia, cartilage loss, and joint destruction [2]. Patients will eventually develop systemic complications that will seriously affect their physical and mental health [3,4]. The most common treatments for RA are nonsteroidal anti-inflammatory drugs (NSAIDs), glucocorticoids (GCS), disease-modifying antirheumatic drugs (DMARDs), and biological agents [5]. However, inflammation and joint damage remain challenging to treat. NSAID inhibits the acetylation of cyclooxygenase to release inflammatory mediators and achieves the purpose of alleviating pain. Loxoprofen (Lox) is a powerful NSAID drug for the treatment of RA [6,7], and the Food and Drug Administration (FDA) has already approved it for use as a topical gel preparation. In addition, tofacitinib (Tof) is a targeted Janus kinase inhibitor [8,9], and oral Tof is a new approach for the treatment of moderate to severe RA in adults who have not responded well to or are intolerant to DMARDs. There was an improvement in the health-related quality of life of RA patients treated with Tof monotherapy (as a first- or second-line treatment) or combination therapy with a conventional synthetic DMARD (csDMARD; as second- or third-line treatment), with sustained benefits following long-term therapy [10]. However, due to the side effects of frequent oral medications on the gastrointestinal tract, the development of approaches that provide better patient compliance and reduce systemic adverse effects is urgently needed [11].

Transdermal delivery systems can avoid the first-pass effect and adverse gastrointestinal effects of oral administration, and, for RA treatment, they have been receiving increasing attention [12]. However, because the stratum corneum (SC) acts as a natural barrier, traditional transdermal drug preparation cannot achieve high skin penetration, and it is difficult to exert the drug’s effect [13]. Microneedles (MNs) are a new type of transdermal drug delivery method and have been widely studied in recent years. They are micro-structured needles with a length of 600–1200 μm [14]. MNs can reversibly pierce the SC and deliver the drug to the dermis of the skin, effectively improving its efficacy [15]. MNs include solid, removable, dissolving, hollow, coated, hydrogel-forming [16], and frozen MNs [17]. Dissolving MNs release drugs after piercing the skin, and they are usually made of biocompatible materials to improve their biological safety [18,19,20]. The natural polymers hyaluronic acid (HA) and chondroitin sulfate (CS) [21], as the main components of human joint synovial fluid [22], are widely used in arthritis treatment and can also be used as materials for preparing dissolving MNs [23,24]. In the treatment of RA, MNs are ideal drug delivery vehicles to directly deliver drugs into the joints [25,26,27,28,29,30,31,32,33].

Photothermal therapy (PTT) uses photothermal materials to convert light energy into heat, thereby killing diseased cells [34], and has widely been applied in the fields of tumor and inflammation treatment in recent years [35]. Similarly to tumor tissues, inflamed tissues are rich in blood vessels from which heat is not easily lost, and they have lower heat resistance than normal tissues [36]. Therefore, PTT can act locally to eliminate inflammatory cells by hyperthermia effects [37]. In addition, drugs can be loaded into delivery systems with photothermal properties and controlled released at the target site by light triggers [38]. Namely, it is possible to combine PTT and drugs on one platform to exert a combined therapeutic effect. Polydopamine (PDA) material is derived from mussel adhesive proteins. It is highly adhesive when wet and a pigment in natural melanin, with good biocompatibility, biodegradability, and photothermal conversion performance [39,40,41,42]. In addition, PDA nanoparticles (NPs) can be formed via the oxidation and self-polymerization of dopamine [43]. 

Based on these advantages of Lox, Tof, MNs, and PDA, we fabricated a PT MN co-loaded with Lox and Tof, with the aim of combining different compositions and functionalities to achieve “all-in-one” therapeutic effects on RA, such as photo-responsive drug release, the MN-mediated permeation enhancement of Lox and Tof, site-directed therapy with the combination of Lox and Tof, and the inhibition of TNF-α (tumor necrosis factor α) and IL-1β (interleukin 1β) biomarkers via PTT. In this study, the Tof is encapsulated into PDA NPs. Then, Tof NPs and Lox are co-loaded into the dissolving MN tips constructed with HA and CS, and the MN pedestal is mainly fabricated with HA and PDA. After MN insertion, followed by light irradiation, the tip can penetrate the SC, pierce through the epidermis, and reach a deeper location. Assisted with the heat induced by light irradiation, the loaded Lox can quickly infiltrate the articular cavity and directly alleviate the pain of arthritis. Meanwhile, the Tof can be released from the PT NPs, achieving controlled release with an 808 nm excitation, and be sustainedly released and retained within the articular cavity to suppress the inflammatory cytokines related to arthritis, heat-boosting the anti-inflammatory effects. The PT MN co-delivery of Lox and Tof is anticipated to synergistically treat RA, with good patient compliance, and RA treatment will be improved by this new approach.

## 2. Materials and Methods

### 2.1. Material

Lox was provided from WEIHAI DISU (WEIHAI DISU, Weihai, China). Tof was purchased from Yibai Biological (Yibai Biological, Beijing, China). Dextran (40 kDa), and dopamine was purchased from Aladdin (Shanghai, China). Macklin Inc. (Shanghai, China) provided *λ*-carrageenan and kaolin. Shangdong Freda Biotechnology Co., Ltd. (Linyi, China) provided hyaluronic acid (HA, 34 kDa, 200–400 kDa). Chondroitin sulfate (CS) was purchased from Dalian Meilun Biological Technology Co., Ltd. (Dalian, China). Sigma-Aldrich (St. Louis, MO, USA) provided rhodamine B (RhB). Gibco (Gaithersburg, MD, USA) provided fetal bovine serum (FBS) and DMEM. Boehringer Mannheim (Paris, France) provided 4′,6-Diamidino-2-phenylindole (DAPI). A local supplier provided all other reagents of analytical grade.

China Center for Type Culture Collection (CCTCC; Wuhan, China) provided HaCaT and Raw264.7 cells.

The Experimental Animal Center of Sun Yat-sen University (Guangzhou, China) provided the Sprague Dawley (SD) rats. During 12 h light/dark cycles, animals were housed under pathogen-free conditions at constant humidity and temperature and were given free access to food and water. Sun Yat-sen University’s Institutional Animal Care and Use Committee approved the protocols. Animals were treated and used in accordance with the Principles of Laboratory Animal Care and Use in Research published by the Chinese Ministry of Health. The number of the approved ethical protocols for this study is SYSU-IACUC-2021-B1284.

### 2.2. Preparation and Characterization of Tof NPs 

Tof NPs were fabricated by the oxidation and self-polymerization method. Briefly, 40 mg of dopamine (DA) was dissolved in 16 mL of pure water as a dopamine solution. Then, 200 μL of ammonia and 4mL of ethanol were added to the dopamine solution with 24 h of agitation. Next, after centrifuging under 12,000 rpm for 15 min, the supernatant was collected. The precipitation was washed with pure water 3 times, and blank PDA NPs were obtained. Then, 20 mg of Tof was added to 20 mL of PDA NP suspension with 24 h of agitation, and the Tof NPs were obtained. 

The NPs were characterized based on their size distribution, zeta potential, and polydispersity index (PDI) by dynamic light scattering (DLS; Malvern Zetasizer Nano-ZS90, Worcestershire, UK). Transmission electron microscopy (TEM; JEM1400 Flash, JEOL, Akishima, Japan) was used to characterize the morphology of the NPs. 

### 2.3. Cellular Experiments

#### 2.3.1. Cellular Uptake 

Raw264.7 cells were cultured at a density of 1 × 10^5^ cells/well with DMEM medium for 12 h. Then, the RhB NP solution, or the RhB solution with an equal concentration of RhB, was added to the cells after the medium was removed. The 808 + RhB NP group was cultured in 808 nm light irradiation (1 W·cm^−2^, 5 min). Then, the precooled PBS was used to wash the wells, and paraformaldehyde was applied at room temperature for 15 min to fix the cells. Then, the wells were washed again, and the cell nuclei were stained by DAPI. A confocal light scanning microscope (CLSM; Olympus FV300, Nagano, Japan) was used to visualize the cellular uptake.

#### 2.3.2. Anti-Inflammatory Effects at the Cellular Level

After different formulations were used to incubate HaCat cells and Raw264.7 cells, their viability was evaluated. The safety of different formulations in vitro was evaluated by MTT (3-(4,5-dimethylthiazol-2-yl)-2,5-diphenyltetrazolium bromide). The different formulations, including blank NPs, a blank MN tip layer mixture, and a blank MN pedestal layer mixture, were diluted 50, 100, and 150 times, respectively, with DMEM. After 24 h of incubation of the different formulations, the MTT solution was added for 4 h. At last, Microplate Readers (FLUOstar Omega-ACU, BioMedical Technology Solutions, Inc., Offenburg, Germany) were used to determine the optical density (OD) of the resultant solution at a wavelength of 490 nm.

Lipopolysaccharide (LPS) was used to irritate Raw264.7 cells to construct the inflammation model at the cellular level. First, a 12-well plate (Corning Inc., Corning, NY, USA) was used to seed the cells. Then, the different formulations, including the Lox solution (100 µg/mL, L100), the Tof solution (10 µg/mL, T10), the mixture of Lox and Tof (L100 + T10), the blank NP solution with 808 nm light irradiation (808 + NPs), the Tof NP solution (10 µg/mL, Tof NPs), the Tof NP solution with 808 nm light irradiation (10 µg/mL, 808 + Tof NPs), and the Tof NP solution and loxoprofen solution with 808 nm light irradiation (808 + Tof NPs + L100), were diluted with DMEM containing 100 ng/mL LPS. Then, the cells were cultured in 808 nm light irradiation (1 W·cm^−2^, 5 min). After 24 h, the total RNA of the cells was isolated by Trizol reagent (Invitrogen, Thermo Fisher Scientific, Inc., Waltham, MA, USA) after being washed, and the PrimeScript RT–PCR kit (Takara, Dalian, China) was used for reverse transcription. The SYBR PrimeScript RT–qPCR kit (Accurate Biotechnology Co, Ltd., Changsha, China) and PCR Cycler (Roche LightCycler 480II, BIOTECON Diagnostics, Potsdam, Germany) were used to perform the quantitative real-time polymerase chain reaction (RT–qPCR). The sequences of the specific primers (Sangon Biotech, Shanghai, China) analyzed for RT–qPCR were listed in Table S1. After 40 cycles of PCR, the expression levels of IL-1*β* and inducible nitric oxide synthase (iNOS) mRNA were calculated using the 2^−ΔΔCt^ relative quantitative method.

### 2.4. Preparation and Characterization of Lox + Tof NPs@MN

#### 2.4.1. Preparation of Lox + Tof NPs@MN

Briefly, 1% (*w*/*v*) Lox and 1% (*w*/*v*) Tof NPs were added to the water with 40% (*w*/*v*) hyaluronic acid (HA) (34 kDa, Freda, China) and 5%, 10%, and 15% (*w*/*v*) CS and mixed thoroughly until the mixture was uniform to obtain a tip-layer matrix solution. In addition, a mixture of 10% (*w*/*v*) HA (34 kDa), 10% (*w*/*v*) HA (200~400 kDa), and 10% (*w*/*v*) dextran40 was added to a 2% (*w*/*v*) PDA suspension and then stirred uniformly to form a pedestal matrix solution. 

Polydimethylsiloxane (PDMS) molds were used to fabricate the Lox + Tof NPs@MN. The fabricated MN patch consisted of 125 needles, and each needle had the shape of a prismatic pyramid, with a height of 1200 μm and a base width of 300 μm. First, 0.080 g of the tip-layer matrix solution was added to the PDMS mold and centrifugated for 3 min at 4000 rpm. Then, the PDMS molds were turned the other way around and centrifugated to the same factor until the mold microcavities were completely filled with the tip-layer solution. Second, 0.350 g of the pedestal matrix solution was applied to the surface of the tip layer, repeating centrifugation at 3000 rpm for 3 min until the pedestal part was paved smoothly. Finally, after drying for 48 h at 4 °C, the MN was finished.

A digital optical microscope (Nikon-Ts2R-FL, Tokyo, Japan) and a scanning electron microscope (SEM, Zeiss EVO MA10, Jena, Germany) were used to observe the appearance morphology of the Lox + Tof NPs@MN.

#### 2.4.2. Mechanical Strength of MN

A displacement–force test station (Force Gauge-Mark-10) was used to test the mechanical strength of the MN patches. First, a single MN patch was horizontally positioned on the platform, with the probe perpendicular to the MN tip. Then, the speed of the sensor probe was set at 1 mm/s to approach the MN tip in the vertical direction. While the sensor probe pressed down on the MN tip, the force and displacement were recorded at a preset displacement of 1200 μm or until the needles broke. 

#### 2.4.3. Insertion Depth of Parafilm and Skin In Vitro

To study the puncture performance of the MN, Parafilm M^®^ was used as a skin simulant [44]. Briefly, ten pieces of parafilm were folded to make a ten-layer film. The MN patch and a pressure-type dynamometer were successively placed on top of the ten-layer film. The pressure-type dynamometer pressed the pedestal of the MN for 5 min, and the force was set to 30 N. Then, both the pressure-type dynamometer and the MN patch were removed, and the number of holes in each layer of film was recorded by microscopy. 

In addition, for further investigating the insertion depth of the MN in the skin, SD rat skin was used as an alternative to human skin. Similarly, the pressure-type dynamometer pressed on the pedestal of MN with force (30 N, 5 min) on the rat skin. Then, the optical coherence tomography (OCT) (TEK SQRAY HSO-2000, Shenzhen, China) was used to record the mean depth and image. After that, hematoxylin and eosin (HE) were used to stain the rat skin, and the skin was observed by microscope.

#### 2.4.4. Morphology of Lox + Tof NPs@MN

Digital optical microscopy (Paulone, XWJ001, Shenzhen, China) was used to examine the morphology of the Lox + Tof NPs@MN, along with a scanning electron microscope (SEM; Quanta 400F; FEI/OXFORD/HKL company, Eindhoven, The Netherlands). For observing the distribution of the MN, an MN was fabricated using RhB NPs instead of Tof NPs with CLSM imaging.

#### 2.4.5. Drug Loading of Lox + Tof NPs@MN

The Lox + Tof NPs@MN were placed in the EP tube and dissolved with distilled water. The above EP tubes were sonicated for 30 min, and the contents of Lox and Tof were determined by high-performance liquid chromatography (HPLC). 

The HPLC system (LC-2030; Shimadzu, Kyoto, Japan) of Lox and Tof was as follows: Both Lox and Tof were analyzed using an XB-C18 column. The mobile phase of Lox was methanol/water (60/40, *v*/*v*, 1.0 mL/min, 40 °C, and 222 nm) [45]. The mobile phase of Tof was acetonitrile/pH 7 buffer (10 mM Na_2_HPO_4_) (25/75, *v*/*v*, 1.0 mL/min, 40 °C, and 286 nm) [46].

#### 2.4.6. The Skin Safety of Lox + Tof NPs@MN

SD rats were treated with the Lox + Tof NPs@MN with 808 nm light irradiation to evaluate the skin safety of Lox + Tof NPs@MN. After treatment for 1 day and 7 days, the TEWL in the knee joint skins were measured, and the treated skins were stained with hematoxylin and eosin (HE) for microscopic observation.

### 2.5. The Photothermal Properties of Tof NPs and Lox + Tof NPs@MN In Vitro 

The influence of PDA on the photothermal properties was investigated with different formulations (blank NPs, Tof NPs, Lox + Tof NPs@MN, and H_2_O) irradiated with 808 nm light (5 min, 1 W·cm^−2^). Blank NPs and Tof NPs in solution (0, 0.5, 1, and 2 mg/mL) were irradiated to study the concentration dependency of the photothermal effect. The blank NPs were irradiated with 808 nm light (0, 0.5, 1.0, and 2 W·cm^−2^) to investigate the power density’s influence. In order to determine the photothermal stability of the NPs, the blank NPs (1 mg/mL) were exposed to ON and OFF for 5 consecutive cycles, each for 5 min. All these experiments were monitored with an IR thermal camera (TiS75 Fluke, Everett, WA, USA) during the irradiation.

### 2.6. Drug Release from Tof NPs and Lox + Tof NPs@MN In Vitro

Tof NPs and Lox + Tof NPs@MN were placed in a 500 Da dialysis bag (MD34MM, DUOXI, Irvine, CA, USA) to investigate the drug release. Then, the dialysis bag was sealed by nylon cable ties and placed into a 50 mL centrifuge tube containing 11 mL of normal saline as a dissolution medium. After that, the 50 mL centrifuge tube was placed on a shaker at 32 °C ± 0.5 °C and shaken at 250 rpm for 12 h. Formulations of 808 + Tof NPs and 808 + Lox + Tof NPs@MN were irradiated at 1 W·cm^−2^ for 5 min. At a preset interval time (15 min, 30 min, and 1, 2, 4, 6, and 8 h), 1 mL of dissolution medium was sampled from the centrifuge tube, and 1mL of normal saline was placed in the centrifuge tube. All the samples were filtered and determined by HPLC to calculate the drug content.

### 2.7. In Vitro and In Vivo Permeation of Tof NPs and Lox + Tof NPs@MN through Rat Skin

Lox + Tof NPs@MN were covered by 3M tape and pressed on the skin with 30 N of force by a dynamometer for 5 min. After being inserted, the skin was installed between the donor and receptor cells (TK-24BL; Kaikai Technology Co, Ltd., Shanghai, China). The NP group donor cells were filled with 500 μL of NP suspension. Formulations of 808 + Tof NPs and 808 + Lox + Tof NPs@MN were irradiated with 808 nm (5 min, 1 W·cm^−2^). The contents of Lox or Tof were the same in each group. All the donor cells were sealed by parafilm during the studies. The receptor cells contained 8 mL of normal saline. The Franz cells were placed in circulating water at 32 °C ± 0.5 °C, and the receiving medium was stirred at 250 rpm. At the time points of 2, 4, 6, 9, 12, and 24 h, the receptor cells were sampled and refilled with fresh medium in an equal volume of 1 mL. After 24 h, the skin was removed and gently wiped three times with a cotton swab and water. Then, the skin was chopped into small pieces, and 2 mL methanol was added for extracting the drugs. The tubes were centrifuged for 30 min, and then 1mL of supernatant was collected. All samples were filtered, and HPLC was used for detection. The infinite dose and occlusion condition were used during permeation studies.

In vivo permeation experiments were performed using SD rats. The SD rats were anesthetized by urethane (20%, *w*/*w*), and the hair of the inner thigh was removed using a shaver and depilatory paste. The SC skin recovered for 24 h, and then MNs were covered with 3 M tape and placed onto the rats’ inner thigh skin. The dynamometer pressed the MNs with 30 N of force for 5 min. For the NP group, 500 μL of Tof NP suspension was applied to the skin. Formulations of 808 + NPs and 808 + MN were irradiated for 5 min at a power density of 1 W·cm^−2^. After 24 h of permeation, the drug retained in the skin was extracted in the same way as in the skin permeation experiments in vitro and measured with HPLC.

### 2.8. Visualization of the Delivery Pathway with CLSM

The RhB NPs’ delivery and distribution of MNs in vitro and in vivo were visualized by CLSM [47]. Using RhB and encapsulating it into NPs to fabricate RhB NPs and RhB NPs@MN, they were used to trace and visualize the delivery of NPs in MN. The abdomen hair of SD rats was removed, and the skin barrier recovered for 24 h. Then, the RhB NPs@ MN were covered by 3M tape and applied onto the inner thigh skin in vitro, and the dynamometer was pressed with 30N for 5 min. For this NP group, donor cells were filled with 500 μL of RhB NP suspension and applied to the skin. Formulations of 808 + RhB NPs and 808 + RhB NPs@MN were irradiated with 808 nm (5 min, 1 W·cm^−2^). After 1 h, the formulations on the skin in vitro were removed and cleaned. Then, the RhB distribution was observed by CLSM scanning, layer by layer. Images were collected from the SC (Z = 0 μm) to the dermis with a spacing of 50 μm. Finally, images of the depth profile were obtained using 3D reconstruction.

The longitudinal RhB NP distribution in the skin in vivo were the same as in in vitro experiments, but all the formulations were applied on the inner thigh skin in vivo for 24 h. Then, all the formulations were removed and cleaned, and the skin was isolated and then cryosectioned at 20 μm spacing. At last, the longitudinal RhB distribution in the skin was observed by CLSM.

### 2.9. Visualization of RhB NPs Distribution in the Joint In Vivo

The SD rats were treated in the same way as in the permeation experiments in vivo. After RhB NPs@MN were stuck to the skin for 1, 2, 4, 6, 9, 12, 18, and 24 h, the legs were dissected, and the skin on the knees was removed at each time point so that the joint was completely presented. An in vivo imaging system (IVISR, NightOWL IILB983, Stuttgart, Germany) was utilized to observe the fluorescence in the joint. A control animal was injected with the same amount of normal saline and RhB intra-articularly. After 24 h, these knees were treated in the same way and observed by IVISR.

### 2.10. Pharmacodynamics Studies

#### 2.10.1. Establishing and Treating Arthritis Models

The model dosage form was a suspension of normal saline containing λ-carrageenan (*w*/*v,* 2%) and kaolin (*w*/*v,* 4%) [48,49]. Briefly, the rats (male, 300–400 g) were anesthetized, and the hair of the inner thigh was removed. The right leg joint was intra-articularly injected with 0.1 mL of the model dosage form, and the left leg was used as the control. After two days, the knee swelling was observed. As follows, 6 groups (n = 5) of arthritic rats were randomly assigned: The model group was given no treatment, and the others were treated with 808 + Blank MN, 808 + Lox@MN, 808 + Tof NPs@MN, 808 + Tof NPs + Lox@Injection, and 808 + Lox + Tof NPs@MN. The 808 + Tof NPs + Lox@Injection group was intra-articularly injected with 0.1 mL Tof NPs + Lox containing the same contents of Lox and Tof with 808 + Lox + Tof NPs@MN on day 2 and day 4.

The diameters of the left and right knee joints in each rat were measured. The swelling degree of the knee joint (%) was calculated using the following formula on days 1, 3, 5, and 7:

$$\mathrm{swelling}\;\mathrm{degree}\;\mathrm{of}\;\mathrm{knee}\;\mathrm{joint}\;(\%)=\frac{\left(\mathrm{right}\;\mathrm{knee}\;\mathrm{diameter}-\mathrm{left}\;\mathrm{knee}\;\mathrm{diameter}\right)}{\;\mathrm{left}\;\mathrm{knee}\;\mathrm{diameter}}\times 100\%$$

Following the euthanasia of the rats on day 7, the gastrocnemius/soleus and tibialis anterior muscles were separated and weighed. The separated articular cartilage was stored at −20 °C until needed, and the muscle-specific gravity (%) was calculated using the following formula to evaluate the degree of atrophy of the lower leg muscles:

$$\mathrm{muscle}\;\mathrm{specific}\;\mathrm{gravity}\;(\%)=\frac{\mathrm{right}\;\mathrm{calf}\;\mathrm{muscle}\;\mathrm{weight}}{\mathrm{left}\;\mathrm{calf}\;\mathrm{muscle}\;\mathrm{weight}}\times 100\%$$

The blood in each rat was collected by the main vein blood sampling method. After 4 h at room temperature, the blood samples were centrifuged for 10 min at 2500 rpm. The supernatant of the serum was collected and stored at −20 °C, and the TNF-*α* was determined by the Enzyme-linked Immunosorbent Assay (ELISA) kit (Abclona, Wuhan, China).

#### 2.10.2. Histopathological Analysis

After the rats were euthanized, the knee joint was separated with a scalpel and scissors and fixed with 4% polyformaldehyde PBS buffer for 24 h. After 3 months of decalcification with EDTA, the wax block of the knee joint was prepared by washing, dehydration, transparency, and embedding. The sample wax was sliced 5 μm thick by a paraffin section machine and then stained with HE and safranin O-fast green. In the end, all of the samples underwent histopathological analysis.

#### 2.10.3. RT-PCR

Using Trizol reagent, the RNA was totally isolated from the articular cartilage (50 mg), and the RT-PCR experiment was similar to that in Section 2.3.2. Table S2 provides the sequences of the RT-qPCR primers. PCR was performed for 50 cycles, and the expression levels of TNF-*α*, IL-1*β*, iNOS, JAK2, JAK3, and STAT3 (signal transducer and activator of transcription 3) mRNA were calculated using the 2^−ΔΔCt^ relative quantitative method.

### 2.11. Statistical Analysis

The data used in the experiments are represented as the mean ± standard deviation and analyzed by one-way ANOVA (SPSS 22.0, SPSS Inc., Chicago, IL, USA) (* *p* ≤ 0.05, ** *p* ≤ 0.01, and *** *p* ≤ 0.001 represent a significant difference).

## 3. Results and Discussions

### 3.1. Physicochemical Characterization of Tof NPs

**Figure 1:**
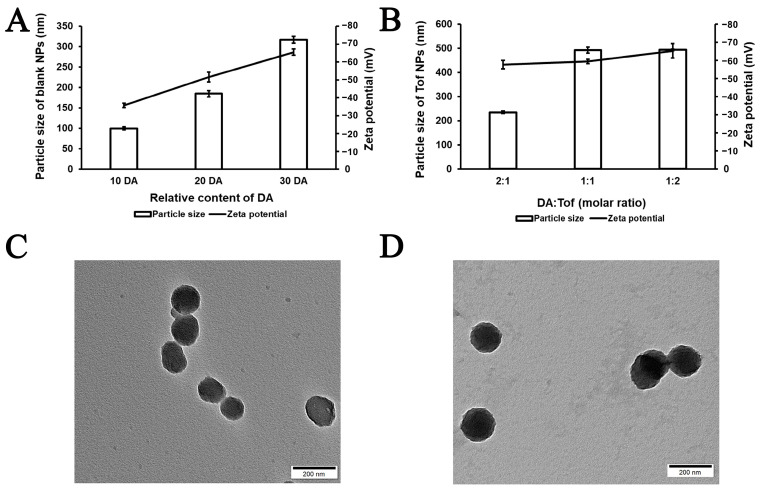
Characterization of Blank NPs and Tof NPs. Screening the amount of DA (**A**); screening the ratio of DA to Tof (**B**); the TEM images of Blank NPs (**C**) and Tof NPs (**D**).

The amount of DA in the blank NPs and the ratio of DA to Tof were screened and are shown in Figure 1A,B. The particle size of the final formulation of the blank NPs was 184.9 ± 7.5 nm, the zeta potential was −51.5 ± 2.7 mV, and the PDI was 0.062 ± 0.027. The particle size of the Tof NPs was 234.2 ± 5.012 nm, the zeta potential was −57.6 ± 2.3 mV, and the PDI was 0.032 ± 0.019. The TEM images of the blank NPs and Tof NPs in Figure 1C,D show that both the blank NPs and Tof NPs had a spherical shape. The size of the blank NPs was approximately 160 nm, and the Tof NPs were approximately 200 nm. As Figure S1 shows, the amount of Tof released from the Tof NPs with 808 nm light irradiation was significantly more than that from the Tof NPs without 808 nm light irradiation. The results of the release experiment suggest that 808 nm could promote drug release from NPs.

### 3.2. Effects of Tof NPs on Raw264.7 Cellular Uptake

**Figure 2:**
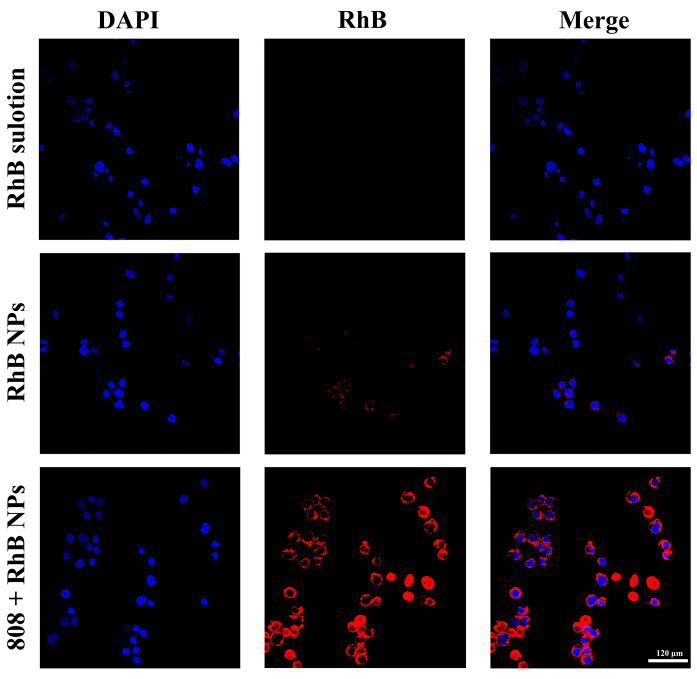
Visualization of RhB uptake into Raw264.7 cells using CLSM after 4 h incubation with different formulations (Scale bar = 120 μm).

Figure 2 shows the RhB NP uptake into Raw264.7 cells using CLSM. Compared to the negligible fluorescence signal in the RhB solution group, the 808 + RhB NP group showed a strong red. The RhB NP group showed a little red but significantly less than the 808 + RhB NP group. This indicates that the NPs could enhance the cellular uptake in Raw264.7 cells, and the photothermal effectof the NPs irradiated by 808 nm light were further promoted the cellular uptake. This result indicated that photothermaleffect could improve the cellular uptake of NPs, which was beneficial to the efficiency of the treatment.

### 3.3. Anti-Inflammatory Effects at the Cellular Level

**Figure 3:**
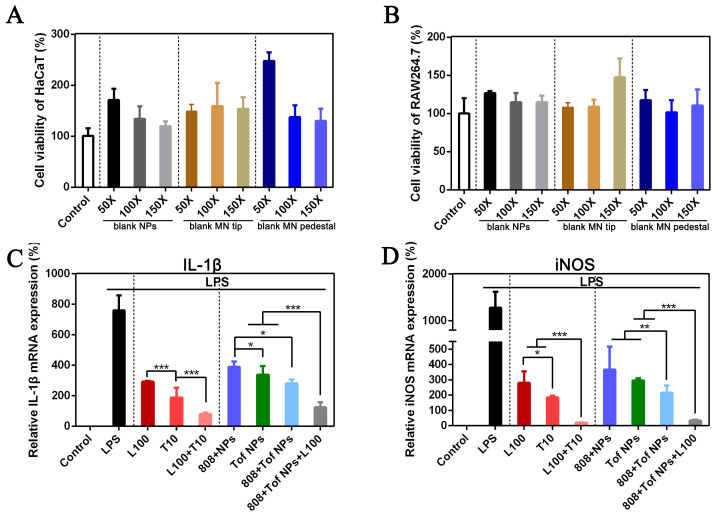
Anti-inflammatory effects at the cellular level. Cell viability of HaCaT (**A**) and Raw264.7 (**B**) cells treated with blank NPs, blank MN tip, and blank MN pedestal. The relative mRNA levels of inflammatory cytokine IL-1*β* (**C**) and iNOS (**D**) after LPS-irritated Raw264.7 cells were treated with different formulations. (* *p* < 0.05, ** *p* < 0.01, and *** *p* < 0.001.)

Figure 3 shows the anti-inflammatory effects at the cellular level. First, the blank NPs and blank MN materials (tip layer and pedestal layer) diluted 50, 100, and 150 times were safe for HaCaT (Figure 3A) and Raw264.7 (Figure 3B) cells, and the cell viability was more than 100%. This is because the materials of the blank NPs and MN were endogenous and biocompatible, such as DA, HA, dextran40, and CS. These biomaterials can promote the proliferation of cells as nutrients. Figure 3C,D show the relative mRNA expressions of IL-1*β* (Figure 3C) and iNOS (Figure 3D) after different treatment formulations. The Lox solution (100 µg/mL) and Tof solution (10 µg/mL) downregulated the inflammatory cytokines IL-1β and iNOS. When Lox was combined with Tof (L100 + T10), the relative mRNA expressions of IL-1*β* and iNOS were significantly lower than those of Lox (L100) or Tof (T10) alone (*p* < 0.001). This proves that both Lox and Tof could inhibit inflammation, and the co-delivery of Lox and Tof inhibited inflammation more significantly. In the final formulations of Lox + Tof NPs@MN, the content ratio of Lox to Tof was 10:1, which exerted the best anti-inflammatory effect. For the IL-1*β* (Figure 3C), the group of 808 + Tof NPs + L100 showed the best downregulation effect compared to the other three groups (808 + NPs, Tof NPs, and 808 + Tof NPs; *p* < 0.001), and the other three groups each showed a different downregulation. The results of the relative mRNA expressions of iNOS (Figure 3D) were similar to those of IL-1*β*, and the group of 808 + Tof NPs + L100 showed the best downregulation effect.

### 3.4. Characterization of Lox + Tof NPs@MN

**Figure 4:**
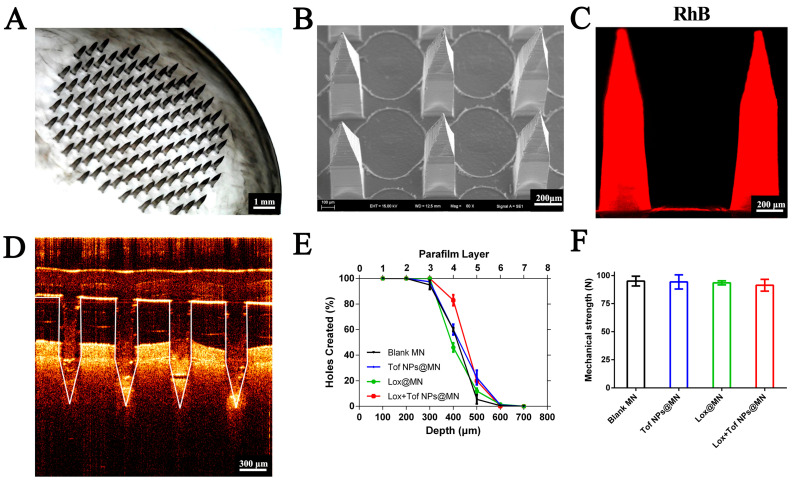
Characterization of MN patches. MN arrays were observed with digital microscopy ((**A**), scale bar = 1 mm), scanning electron micrography ((**B**), scale bar = 200 μm), and confocal micrograph of MN ((**C**), scale bar = 200 μm). OTC images of MN insertion into skin ((**D**), scale bar = 300 μm), insertion depth (**E**), and mechanical strength (**F**) of different MNs.

The appearance of Lox + Tof NPs@MN was characterized by digital microscopy and SEM. The Lox + Tof NPs@MN patch consisted of 125 consecutive and intact needles (Figure 4A), and each needle had a pyramid prismatic shape (Figure 4B). The RhB NPs replaced the Tof NPs to fabricate the RhB NPs@MN, as shown in Figure 4C by CLSM, and red RhB fluorescence was distributed throughout the needle, which proves that the NPs in the needle were evenly distributed.

To screen the content of CS in the needle, we investigated the insertion depth (Figure S2A) and mechanical strength (Figure S2B) of different formulations (containing 5%, 10%, and 15% CS *w*/*w*) of MNs. In the MN with 5% CS, the puncture pore ratio was 100 ± 0.0% for the first, second, third layer; 98.4 ± 2.3% for the fourthlayer; and 68.0 ± 4.7% for the fifth layers. The MN containing 5% CS could penetrate the ten-layer film with the highest puncture pore ratio, which indicates that the insertion depth in the skin was up to approximately 500 μm. In addition, the mechanical strength in all of the different formulations (5%, 10%, and 15% CS) of MNs was more than 90 N/patch (0.72 N/needle, Figure S2B). Therefore, the final content of CS was 5% (*w*/*w*) in the tip-layer matrix solution.

Using OCT (Figure 4D), the Lox + Tof NPs@MN patch, together with the treated skin, was imaged to determine the insertion depth of the MN in the skin. The insertion depth was 450–500 µm, as observed by OCT, which indicates that the MN can penetrate both the epidermis and dermis of the skin. Meanwhile, the HE-stained sections are shown in Figure S3. The MN-treated skin presented obvious puncture marks, and the insertion depth was ~400 µm after MN treatment. The results observed by OCT and the HE-stained sections are mutually corroborated by the results of the insertion depth in the ten-layer film.

Furthermore, the blank MN, Tof NPs@MN, Lox@MN, and Lox + Tof NPs@MN were fabricated for treating arthritis, and the insertion depth (Figure 4E) and mechanical strength (Figure 4F) of these MNs were investigated. All of the MNs could reach the fifth layer with their insertion depth, and they had more than 90 N/patch of mechanical strength.

The drug loading of each Lox + Tof NPs@MN patch was as follows: the Lox was 494.99 ± 6.15 μg/patch, and the Tof was 36.84 ± 4.14 μg/patch. The drug loadings relative to the initial amounts added were 61.9% (Lox) and 54.8% (Tof) separately. The proportions of the two drugs are consistent with the proportions of clinical dosages.

The HE-stained sections before/after the MN treatment are shown in Figure S4. There was no obvious inflammatory cell infiltration in the HE-stained sections before the MN treatment (day 0) or after the MN treatment (days 1 and 7). In addition, the TEWL after different treatments is shown in Figure S5. After the MN treatment, the TEWL increased in the first 30 min but then decreased and was consistent with the control group within an hour. The results of the 808 + Lox + Tof NPs@MN group were the same as those of the Lox + Tof NPs@MN group. Lox + Tof NPs@MN with 808 nm light treatment was safe on the SD rat skin.

The amounts of Lox and Tof released from the Lox + Tof NPs@MN are shown in Figure S6. The amount of Lox released from the 808 + Lox + Tof NPs@MN was similar to that from the Lox + Tof NPs@MN without 808 nm light. However, the amount of Tof released from 808 + Lox + Tof NPs@MN was more than that from the Lox + Tof NPs@MN without 808 nm light. The results show that the release of Tof encapsulated by NPs in the MN still responded to the photothermal action, whereas Lox directly loaded in the MN was not greatly affected by photothermal action.

### 3.5. The Photothermal Properties of Tof NPs and Lox + Tof NPs@MN In Vitro

**Figure 5:**
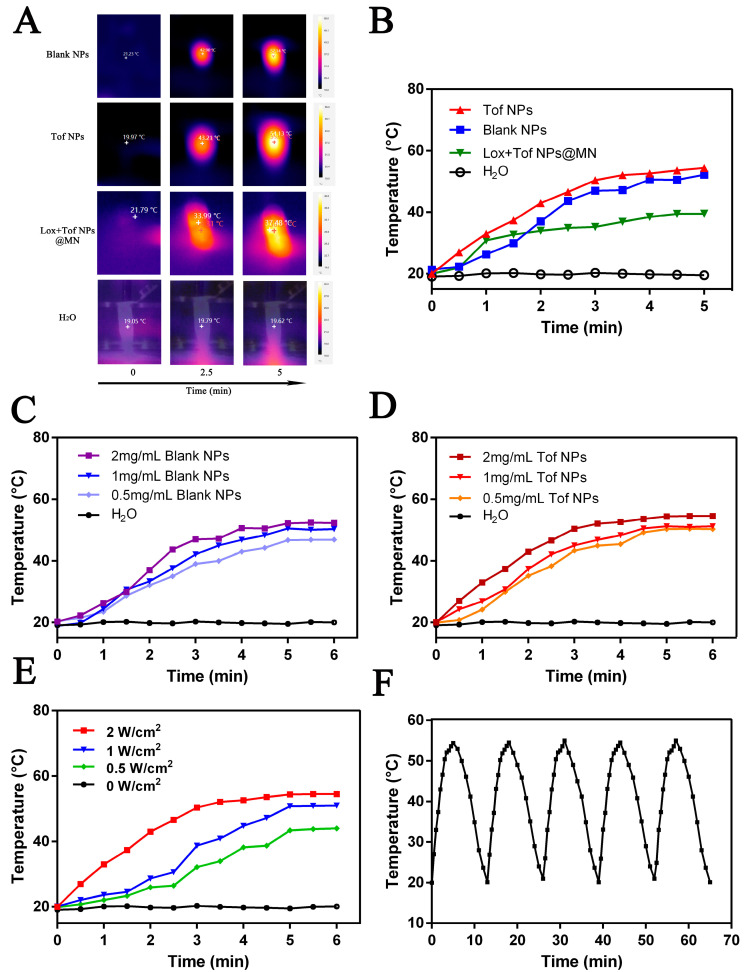
Characterization of the photothermal properties of NPs and MN patches in vitro. The photothermal images of Blank NPs, Tof NPs, and Lox + Tof NPs@MN (**A**); photothermal curves of different formulations with light irradiation (**B**); photothermal curves of Blank NPs and Tof NPs with different concentration with light irradiation (**C**,**D**); photothermal curves of blank NPs irradiated at various power densities of 0, 0.5, 1, and 2 W·cm^−2^ (**E**); heating/cooling profiles of blank NPs for five repeated intervals of 5-min-on and 5-min-off light irradiations (**F**). The irradiation was generally performed with an 808 nm near-infrared light at the power density of 1 W·cm^−2^ for 5 min.

The in vitro photothermal performance of Tof NPs and Lox + Tof NPs@MN are shown in Figure 5. The photothermal images were recorded after different formulations were irradiated using a power density of 1 W·cm^−2^ for 5 min with an 808 nm light (Figure 5A). There was obviously photothermal performance in the blank NP and Tof NP suspensions; the suspension temperature rose to 50 °C within 5 min (Figure 5B). Interestingly, the temperature of Lox + Tof NPs@MN rose to 40 °C within 5 min and remained stable. This may have been because the HA, CS, and PDA in the MN pedestal were in a certain proportion, which reduced the overall photothermal properties of Lox + Tof NPs@MN. The temperature of 40 °C was mild and exerted photothermal performance without irritating the skin. Therefore, the irradiation of 808 nm (5 min, 1 W·cm^−2^) was a condition of the treatment administering Lox + Tof NPs@MN. The photothermal effect varied with different concentrations of the blank NP and Tof NP solutions and is shown in Figure 5C,D, respectively. There was an obvious concentration dependency of the blank NP and Tof NP solutions (0, 0.5, 1, 2 mg/mL). In addition, as the light power density increased (0, 0.5, 1.0, and 2 W·cm^−2^; Figure 5E), the photothermal performance of the blank NP solution gradually increased. The NP and blank NP solutions (1 mg/mL) were irradiated for 5 min on and 5 min off five times to determine their photothermal stability (Figure 5F). The results show that there was no obvious temperature decay after five cycles of light on/off for the blank NPs. In short, the photothermal performance of the blank NPs was excellent, and there was no significance between the Tof NPs and the blank NPs.

### 3.6. In Vitro Permeation of Tof NPs and Lox + Tof NPs@MN through Rat Skin

To assess the delivery behavior of Tof and Lox by the Tof NPs and Lox + Tof NPs@MN, the Tof solution, Tof NPs, 808 + Tof NPs, Lox + Tof NPs@MN, and 808 + Lox + Tof NPs@MN groups were examined in in vitro permeation studies.

**Figure 6:**
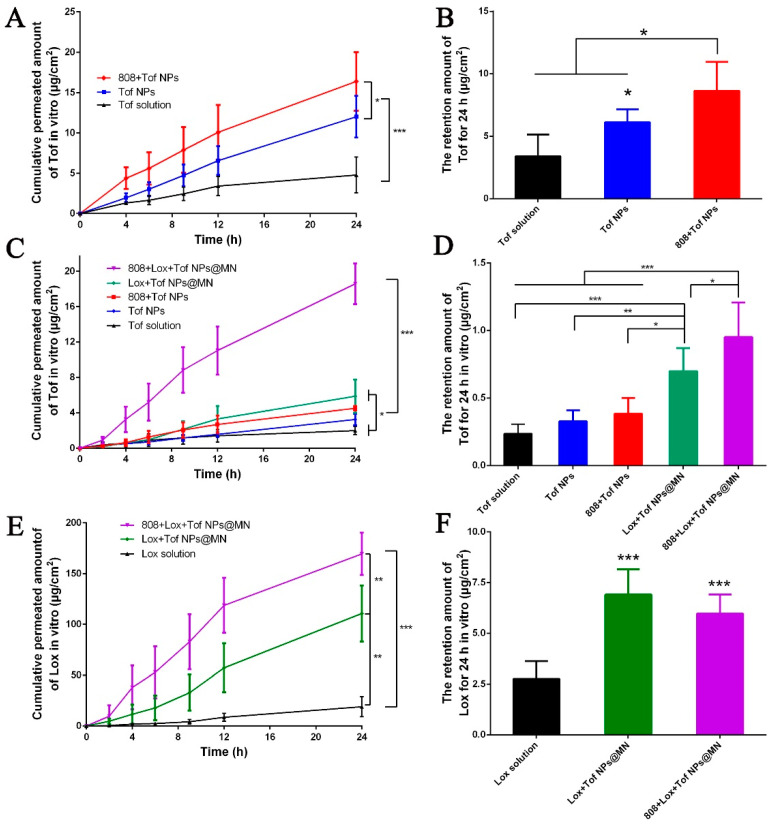
In vitro permeation of Tof NPs and Lox + Tof NPs@MN through rat skin for 24 h. Cumulative permeation (**A**) and skin retention (**B**) of Tof from Tof NPs with or without light irradiation. Cumulative permeation (**C**) and skin retention (**D**) of Tof from Lox + Tof NPs@MN with or without light irradiation. Cumulative permeation (**E**) and skin retention (**F**) of Lox from Lox + Tof NPs@MN with or without light irradiation. (n = 6, * *p* < 0.05, ** *p* < 0.01, and *** *p* < 0.001).

The cumulative penetration amount of Tof and the skin retention of Tof NPs are shown in Figure 6A,B, respectively. The 24 h cumulative penetration amount of Tof in the 808 + Tof NP group was 16.38 ± 3.64 μg·cm^−2^, which was significantly higher than that of the Tof NP group (12.01 ± 2.59 μg·cm^−2^, *p* < 0.05) and the Tof solution group (4.79 ± 2.23 μg·cm^−2^, *p* < 0.001). The cumulative penetration amount of Tof in the 808 + Tof NP group was 3.4 times that of the Tof solution and 1.3 times that of the Tof NP group. The skin retention of the Tof in vitro is shown in Figure 6B. The Tof retention levels in the skin of the 808 + Tof NP group, the Tof NP group, and the Tof solution were 8.64 ± 2.32, 6.12 ± 1.02, and 3.41 ± 1.74 μg·cm^−2^, respectively. The skin retention in the 808 + Tof NP group was significantly higher than that in the Tof NP group (*p* < 0.05) and the Tof solution group (*p* < 0.05). The combination of Tof NPs and photothermal effects could synergistically promote transdermal drug delivery.

After 24 h, the amounts of the cumulative penetration of Tof (Figure 6C,D) and Lox (Figure 6E,F) and the skin retention in the Tof NPs and Lox + Tof NPs@MN groups were determined. The amounts of Tof penetration in the 808 + Lox + Tof NPs@MN group, the Lox + Tof NPs@MN group, the 808 + Tof NP group, the Tof NP group, and the Tof solution group were, respectively, 18.57 ± 2.29, 5.88 ± 1.87, 4.51 ± 0.25, 3.25 ± 0.65, and 1.99 ± 0.48 μg·cm^−2^ (Figure 6C). The cumulative penetration amounts of Tof in the 808 + Lox + Tof NPs@MN group was 3.1 times that of the Lox + Tof NPs@MN group (*p* < 0.001) and significantly higher than the other groups (*p* < 0.001), and the penetration amount in the Lox + Tof NPs@MN group was 2.9 times that in the Tof solution group (*p* < 0.05). Figure 6D shows the skin retention of Tof in vitro. The skin retention levels of the 808 + Lox + Tof NPs@MN group, the Lox + Tof NPs@MN group, the 808 nm + Tof NP group, the Tof NP group, and the Tof solution group were, respectively, 0.86 ± 0.30, 0.62 ± 0.24, 0.38 ± 0.12,0.31 ± 0.08, and 0.23 ± 0.07 μg·cm^−2^. The retention of Tof in the skin in the 808 + Lox + Tof NPs@MN group was significantly higher than that in the Lox + Tof NPs@MN group (*p* < 0.05) and the other groups (*p* < 0.001).

The amounts of Lox penetration in the 808 + Lox + Tof NPs@MN group, the Lox + Tof NPs@MN group, and the Lox solution group were, respectively, 169.42 ± 20.83, 110.72 ± 27.67, and 19.01 ± 9.96 μg·cm^−2^ (Figure 6E), and the 808 + Lox + Tof NPs@MN group had higher amounts than the Lox + Tof NPs@MN group (*p* < 0.01) and significantly higher amounts than the Lox solution group (*p* < 0.001). The Lox skin retention levels of the 808 + Lox + Tof NPs@MN group, the Lox + Tof NPs@MN group, and the Lox solution group were, respectively, 6.90 ± 1.25, 5.79 ± 0.93, and 2.76 ± 0.87 μg·cm^−2^ (Figure 6F), and that of the 808 + Lox + Tof NPs@MN group was close to that of the Lox + Tof NPs@MN group (*p* > 0.05) but significantly higher than that of the Lox solution group (*p* < 0.001). Interestingly, as observed in the skin penetration study of 808 + Lox + Tof NPs@MN, the cumulative penetration of Lox was very high, indicating that 808 + Lox + Tof NPs@MN could deliver more Lox to the deep skin and treat arthritis. The cumulative penetration and retention of Tof were also significantly higher than in the other groups, indicating that 808 + Lox + Tof NPs@MN could provide fast and sustained anti-inflammatory action. The achieved drug penetration rates are shown in Figure S7.

### 3.7. Visualization of the Delivery Pathway with CLSM

**Figure 7:**
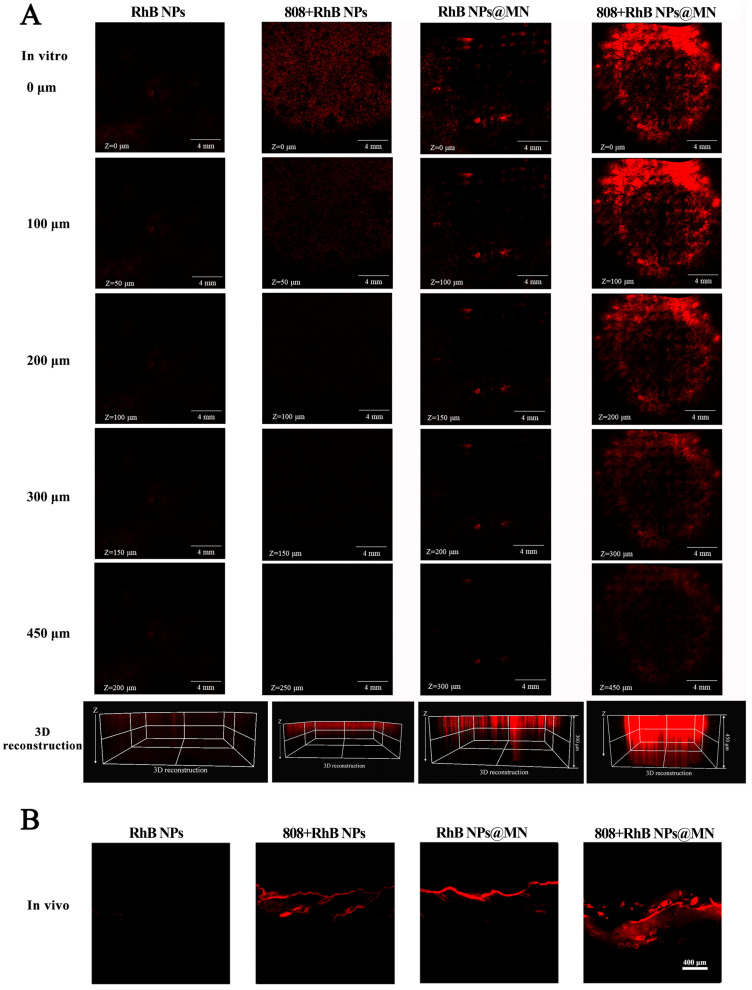
Visualization of RhB NPs’ permeation in the skin from different formations with CLSM in vitro (**A**) and in vivo (**B**).

RhB NPs are rapidly distributed from the skin to the joints, which is very important for arthritis treatment. The RhB NPs distribution, layer by layer in the skin over time, was observed by CLSM (Figure 7A), and the CLSM micrographs show the skin sections after the RhB NPs, 808 + RhB NPs, RhB NPs@MN, and 808 + RhB NPs@MN applications of 60 min. It is clearly shown that the red fluorescence signal near the skin epidermis (z-position = 0 μm) was stronger than that of the deep skin (z-position = 450, 300, 250, and 200 μm) in the four groups. Moreover, it can be clearly observed that the red fluorescence signal in the 808 + RhB NPs@MN was the strongest and deepest (z-position = 450), and the MN group was stronger and deeper than the RhB NPs + 808 group. This once again proves that the RhB NPs were delivered deep into the skin due to the MN, and its combination with photothermal action further improved the ability of the MN to deliver drugs to the skin.

The skin sections after the 24 h applications of RhB NPs, 808 + RhB NPs, RhB NPs@MN, and 808 + RhB NPs@MN are shown in Figure 7B. The red fluorescence signal in the 808 + RhB NPs@MN group was significantly deeper than that in the other groups. This result is corroborated by the result of the in vivo skin retention (Figure S8). In Figure S8, the in vivo skin retention levels of Tof in the Tof NP, 808 + Tof NP, Lox + Tof NPs@MN, and 808 + Lox + Tof NPs@MN groups were, respectively, 0.05 ± 0.02, 0.08 ± 0.04, 0.14 ± 0.03, and 0.20 ± 0.02 μg·cm^−2^, and those in the 808 + Lox + Tof NPs@MN group were markedly higher than those in the MN group (*p* < 0.01) and the other groups (*p* < 0.001). The visualization and quantification of the skin retention studies demonstrate that the 808 nm light irradiation significantly enhanced the NPs’ permeation and retention.

### 3.8. Visualization of RhB NPs Distribution in the Joint In Vivo

**Figure 8:**
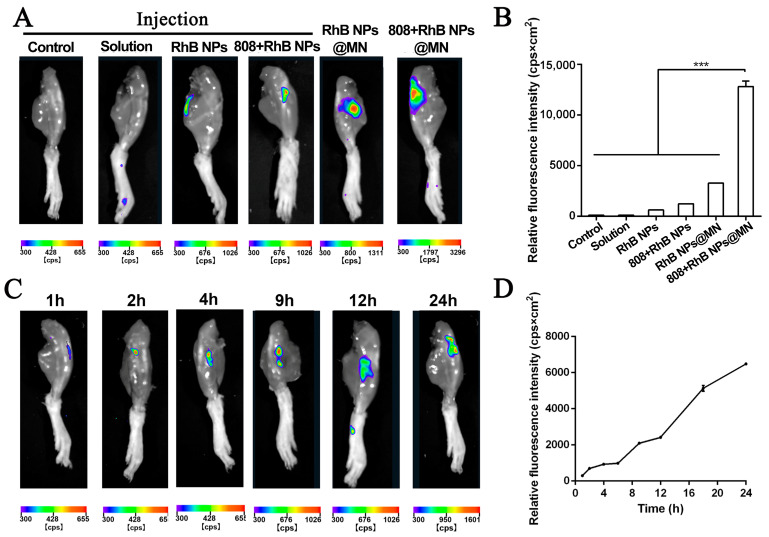
In vivo images of RhB retention in the joint of rats after being administered with different formulations (**A**) and the relative fluorescence intensity (**B**). In vivo images of RhB retention in the articular cavity variation with the time up to 24 h after MN administration with light irradiation (**C**) and the relative fluorescence intensity (**D**). *** *p* < 0.001.

The IVISR was used to visualize the RhB NP distribution in the SD rat knee joint (Figure 8) to confirm whether the RhB NPs loaded in the MN could penetrate the skin and diffuse into the joint. The rat knee joint images after being punctured by the RhB NPs@MN or 808 + RhB NPs@MN, an intra-articular injection with an RhB solution, RhB NPs, and 808 + RhB NPs are shown in Figure 8A, and the relative fluorescence is shown in Figure 8B. The different formulations (RhB solution, RhB NPs, 808 + RhB NPs, RhB NPs@MN, and 808 + RhB NPs@MN) loaded with RhB were applied to the knee joint for 24 h. The fluorescence intensity was the strongest in the 808 + RhB NPs@MN group, which proves that the photothermal effect further improved the RhB NP accumulation in the joint cavity. The accumulation levels in the joint of the intra-articular injection with the RhB solution, RhB NPs, and 808 + RhB NPs were lower, and this may have been because the injected systemic distribution and metabolization were rapid. After the 808 + RhB NPs@MN treatment had been applied to the knee joint for 1, 2, 4, 9, 12, and 24 h, the RhB red fluorescence was observed, and it increased gradually with time, reaching the highest level at 24 h (Figure 8C,D). Based on the above results, we speculated that the light (808 nm) irradiation and MN enable drugs to reach the joint, and therefore, the treatment time of the 808 + Lox + Tof NPs@MN was set to 24 h.

### 3.9. Effects of Lox + Tof NPs@MN on Rheumatoid Arthritis Treatment

#### 3.9.1. Observations on Anti-Arthritis

**Figure 9:**
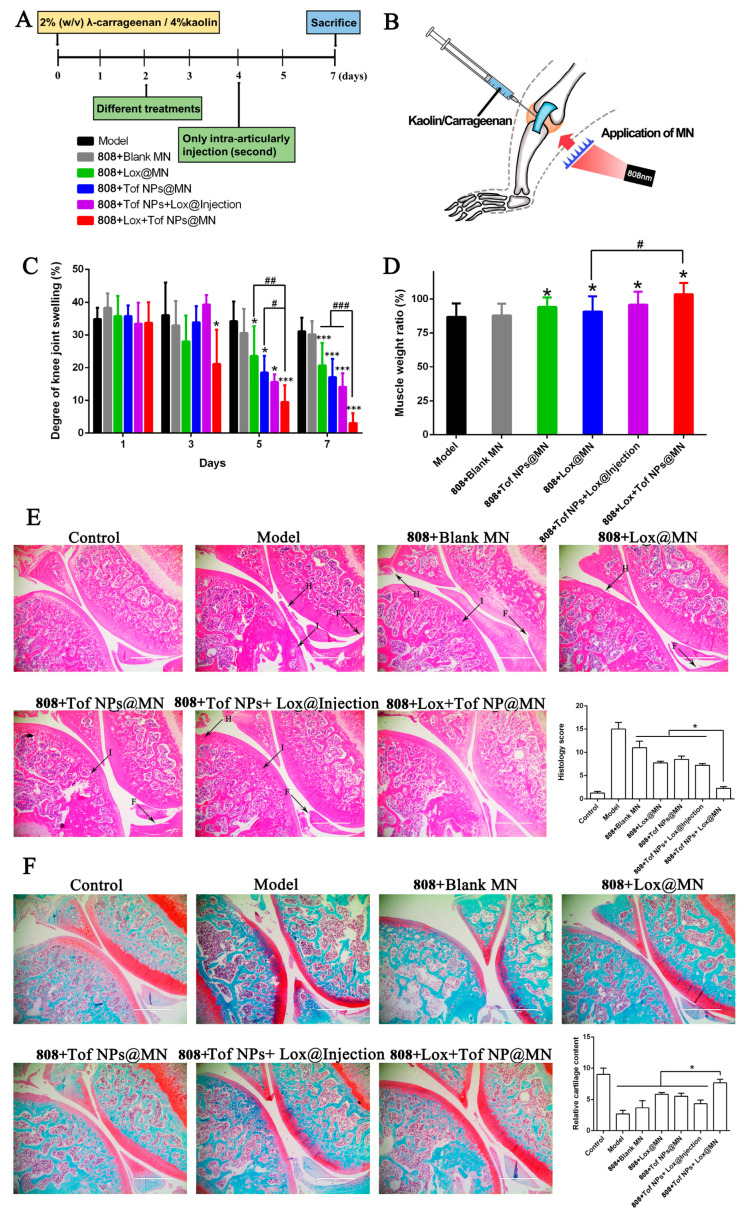
Effects of different formulations on arthritis treatment. The schematic diagram of protocols for arthritis rat modeling (**A**) and treating (**B**). Degree of knee swelling (**C**) and atrophy of crus muscle (**D**). Histological features of knee joint stained with HE ((**E**), letters H, I, F with black arrows respectively represent synovium hyperplasia, immune cell infiltration, and synovial membrane fibrillation) and safranin O-fast green (**F**) in arthritis rats (magnification 100×; scale bar: 1000 μm) (n = 5; * *p* < 0.05, and *** *p* < 0.001; ^#^ *p* < 0.05, ^##^ *p* < 0.01, and ^###^ *p* < 0.001).

To evaluate its therapeutic effects on anti-arthritis in SD rats, the model group, the 808 + Blank MN group, the 808 + Lox@MN group, the 808 + Tof NPs@MN group, the 808 + Tof NPs + Lox@Injection group, and the 808 + Lox + Tof NPs@MN group were set up. In Figure 9A,B, schematic diagrams show how the arthritis models were established and how the treatment protocol was designed. All the groups were irradiated with 808 nm light (5 min, 1 W·cm^−2^). As Figure 9C shows, on the first day after modeling, the knee of the model group swelled rapidly, and the degree of knee swelling reached 34.80 ± 3.50%. The knee swelling was alleviated to varying degrees after treatment with different formulations. On day 7 post-treatment, the knee swelling in the model, 808 + Blank MN, 808 + Lox@MN, 808 + Tof NPs@MN, 808 + Tof NPs + Lox@Injection, and 808 + Lox + Tof NPs@MN groups were, respectively, 31.10 ± 4.10%, 30.20 ± 4.00%, 20.70 ± 6.90%, 17.10 ± 5.60%, 14.10 ± 4.20%, and 3.10 ± 3.00%. Encouragingly, the knee swelling in the 808 + Lox + Tof NPs@MN group was significantly lower than that in the other treatment groups (*p* < 0.001).

In addition, the calf muscle weight was measured in all groups. As Figure 9D shows, the proportions of calf muscle mass in the model, 808 + Blank MN, 808 + Lox@MN, 808 + Tof NPs@MN, 808 + Tof NPs + Lox@Injection, and 808 + Lox + Tof NPs@MN groups were, respectively, 86.76 ± 9.95%, 87.80 ± 8.67%, 94.13 ± 7.00%, 90.64 ± 11.34%, 95.82 ± 9.58%, and 103.48 ± 8.31%. The treatment groups alleviated calf muscle atrophy to varying degrees, and the treatment effect of the 808 + Lox + Tof NPs@MN group was the most obvious compared to the model group (*p* < 0.05).

#### 3.9.2. Histopathological Analysis

As shown in Figure S9, the serum TNF-*α* was measured after the rats were euthanized. The serum TNF-*α* content in the 808 + Lox + Tof NPs@MN group was the lowest compared to the other treatment groups (*p* < 0.05) and the model group (*p* < 0.001). This indicates that the treatment of 808 + Lox + Tof NPs@MN at the knee joint site can make the drug enter the blood and downregulate the inflammatory factors of TNF-*α*. Interestingly, the downregulation of TNF-*α* in the 808 + Lox + Tof NPs@MN group was two times better than with the intra-articular treatment and the other MN treatments.

In the animal experiment, after 7 days, the knee joint samples in all the groups underwent histopathological analysis. As Figure 9E shows, in the control group, the surface of the knee joint cartilage was smooth, the thickness was uniform, and the chondrocytes were normal in shape and arranged regularly. There was no infiltration of inflammatory cells in the joint cavity. For the model group, the surface of the articular cartilage was rough, the arrangement of chondrocytes was disordered, and lots of inflammatory cells infiltrated the synovial tissue, showing symptoms of synovitis. The treatments with different formulations relieved the symptoms of arthritis to some extent. Encouragingly, in the 808 + Lox + Tof NPs@MN group, the chondrocytes were regularly arranged and calcified evenly, without an obvious infiltration of inflammatory cells. The histology scores [27] show that 808 + Lox + Tof NPs@MN significantly alleviated the fibrillation of the synovial membrane and hyperplasia of the synovium, and the infiltration of immune cells are symptoms of this condition.

Furthermore, safranin O–fast green was used to stain the knee joint sections of each group to observe the conditions of the articular cartilage. The basic dye safranin O can be combined with basophilic cartilage to be red, and the acid dye fast green can be combined with eosinophilic bone tissue to be green or blue; that is, red is cartilage and blue-green is the bone tissue. As Figure 9F shows, the articular cartilage of the model group was severely damaged, and a large area of safranin was lost, indicating that the cartilage matrix proteoglycan degraded. The different MN groups were analogous, and the safranin loss decreased, indicating that the cartilage damage was alleviated. Importantly, the 808 + Lox + Tof NPs@MN group showed the best cartilage recovery.

#### 3.9.3. RT-qPCR

**Figure 10:**
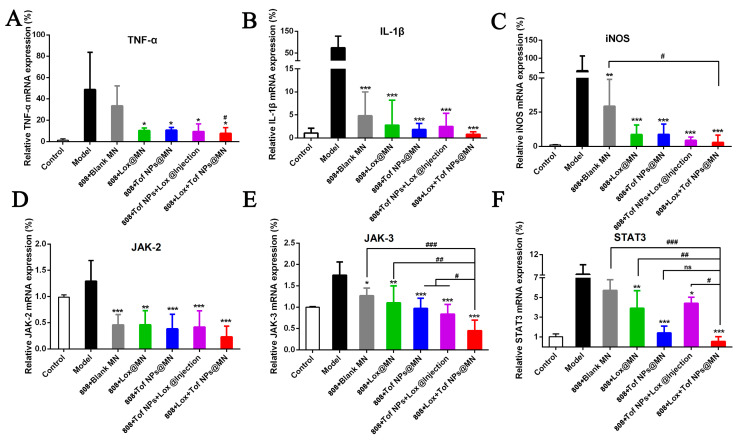
Effects of different MN formulations on the relative mRNA levels of inflammatory cytokines in the knee cartilage of rat. TNF-*α* (**A**); IL-1*β* (**B**); iNOS (**C**); JAK2 (**D**); JAK3 (**E**); STAT3 (**F**). (n = 4; * *p* < 0.05, ** *p* < 0.01, and *** *p* < 0.001; ^#^ *p* < 0.05, ^##^ *p* < 0.01, and ^###^ *p* < 0.001; ns means no significance).

The articular cartilage was isolated, and RT-PCR was performed to analyze its inflammation. Figure 10A–F show the relative mRNA levels of inflammatory cytokines in the articular cartilage. The relative mRNA expressions of TNF-*α* (A), IL-1*β* (B), and iNOS (C) had the same trend, and the mRNA expression’s downregulation in the 808 + Lox + Tof NPs@MN group was the most evident compared to the other treatment groups. The treatment groups, including the blank MN group, showed varying degrees of the downregulation of IL-1*β* and iNOS. It was shown that the MN combined with the photothermal treatment effectively downregulated the inflammation level of the cartilage in the arthritis model. To further verify the anti-inflammatory mechanism of Tof, the relative mRNA expressions of JAK2(D), JAK3(E), and STAT3(F) were analyzed. The downregulation in the 808 + Lox + Tof NPs@MN group was the most remarkable compared to the other treatment groups, especially for JAK3 and STAT3. Similarly, the other treatment groups, including the 808 + Blank MN group, showed varying degrees of the downregulation of JAK2, JAK3, and STAT3, especially the groups that contained Tof. Importantly, the 808 + Blank groups showed the downregulation of proinflammatory cytokines, which was due to the HA and CS materials and their combination with the photothermal treatment.

## 4. Conclusions

RA is an autoimmune disease of synovial inflammation. In this work, for the first time, we developed an MN with photothermal properties, which co-delivers Tof and Lox for RA treatment. In vitro and in vivo skin permeation studies demonstrated that the Lox + Tof NPs@MN treatment with 808 nm light irradiation significantly enhanced the drug permeation and retention in the skin and the articular cavity. The therapeutic efficacy of 808 + Lox + Tof NPs@MN was even two times better than the intra-articular treatment against arthritis. The 808 + Lox + Tof NPs@MN downregulated the mRNA levels of inflammatory cytokines, including TNF-*α*, IL-1*β*, iNOS, JAK2, JAK3, and STAT3, in the articular cartilage. In conclusion, the results indicate that the 808 + Lox + Tof NPs@MN system provides a convenient and potential strategy for RA treatment.

## Data Availability

The data presented in this study are available from the corresponding author on reasonable request.

## Supplementary Materials

The following supporting information can be downloaded at: https://www.mdpi.com/article/10.3390/pharmaceutics15051500/s1, Figure S1. In vitro release of Tof from Tof NPs with or without light irradiation. Figure S2. Screening the content of CS in MN formulation. Figure S3. The HE stained sections before and after 808 + Lox + Tof NPs@MN treatment. Figure S4. The HE stained sections before 808 + Lox + Tof NPs@MN treatment (Day 0) and after 808 + Lox + Tof NPs@MN treatment (Day 1 and Day 7). Figure S5. The TEWL after different treatment (Control, Lox + Tof NPs@MN, and 808 + Lox + Tof NPs@MN). Figure S6. In vitro release of Tof and Lox from Lox + Tof NPs@MN. Cumulative release rate of Lox and Tof from Lox + Tof NPs@MNs with or without light irradiation. The irradiation was performed with 808 nm near infra-red light at the power density of 1 W·cm^−2^ for 5 min prior to in vitro release. Figure S7. In vitro permeation of Tof NPs and Lox + Tof NPs@MN through rat skin for 24 h. Figure S8. Skin retention of Tof for 24 h in vivo permeation from different formulations. Figure S9. The serum TNF-α of arthritic rats. Table S1: DNA sequence of primer pairs used for quantitative real time PCR in cells. Table S2: DNA sequence of primer pairs used for quantitative real time PCR in rats.

## Abbreviations

CLSM, confocal laser scanning microscope; CS, chondroitin sulfate; DA, dopamine; DAPI, 4′,6-diamidino-2-phenylindole; DMARDs, disease-modifying antirheumatic drugs; ELISA, enzyme-linked immunosorbent assay; FDA, Food and Drug Administration; HA, hyaluronic acid; HE, hematoxylin–eosin staining; HPLC, high-performance liquid chromatography; IL-1*β*, interleukin 1*β*; iNOS, inducible nitric oxide synthase; IVISR, in vivo imaging system; JAK2, Janus kinase 2; JAK3, Janus kinase 3; Lox, loxoprofen; Lox@MN, MNs loading Lox only; Lox + Tof NPs@MN, MNs loading Tof NPs and Lox; LPS, Lipopolysaccharide; MN, microneedle; MTT, 3-(4,5-dimethylthiazol-2-yl)-2,5-diphenyltetrazolium bromide; NSAIDs, non-steroidal anti-inflammatory drugs; PT, photothermal; PTT, photothermal therapy; PDA, polydopamine; PDI, polydispersity index; PDMS, polydimethylsiloxane; OCT, optical coherence tomography; qRT-PCR, quantitative real-time polymerase chain reaction; RA, rheumatoid arthritis; RhB, rhodamine B; SD, Sprague Dawley; SEM, scanning electron microscope; SC, stratum corneum; STAT3, signal transducer and activator of transcription 3; TEM, transmission electron microscopy; TNF-*α*, tumor necrosis factor *α*; Tof, tofacitinib; Tof NPs, tofacitinib-loaded nanoparticles; Tof NPs@MN, MN-loaded Tof NPs only.
