# Supplementary material for: Co-Delivery of Loxoprofen and Tofacitinib by Photothermal Microneedles for Rheumatoid Arthritis Treatment
Source: Pharmaceutics. 2023 May 15;15(5):1500. doi: 10.3390/pharmaceutics15051500 (PMC10220954; doi:10.3390/pharmaceutics15051500)
Supplement: Supplementary file 1 [file pharmaceutics-15-01500-s001.zip › pharmaceutics-2277289-supplementary.pdf]

Supporting Information

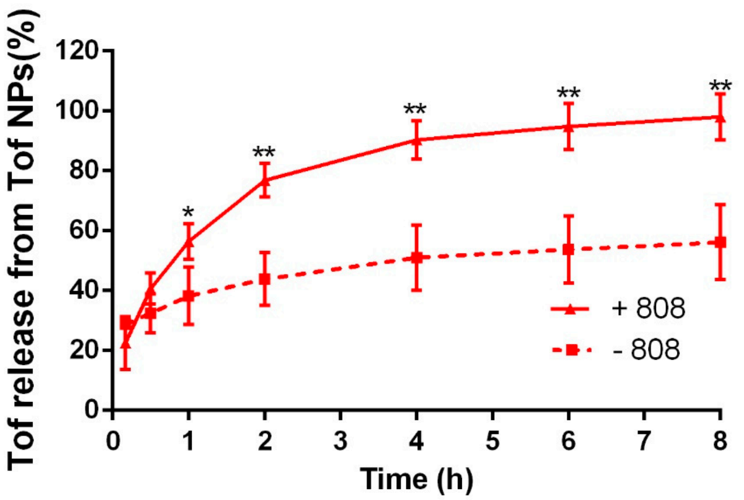

Figure S1. *In vitro* release of Tof from Tof NPs with or without light irradiation. The irradiation was performed with 808 nm near infra-red light at the power density of 1 $W \cdot cm^{-2}$ for 5min prior to *in vitro* release. (*$p < 0.05$, **$p < 0.01$, and ***$p < 0.001$).

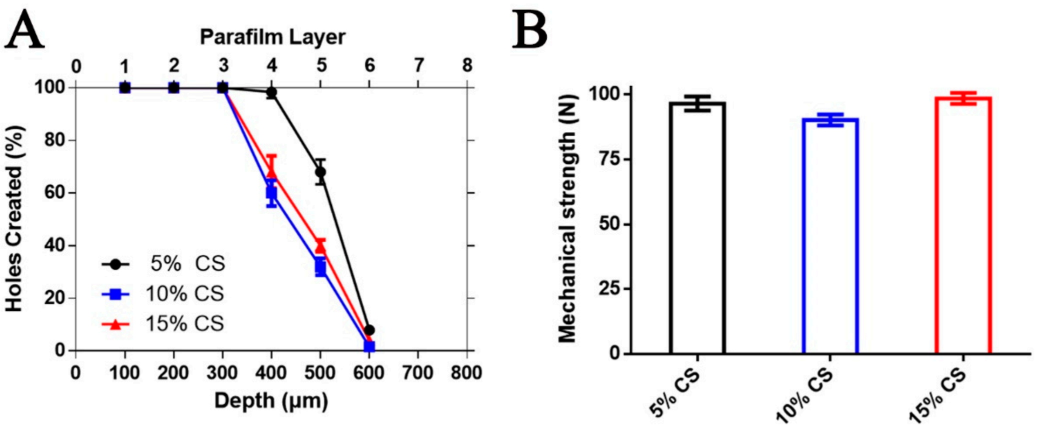

Figure S2. Screening the content of CS in MN formulation. The insertion depth (A), mechanical strength (B) of different (containing 5%,10%,15% of CS) MNs.

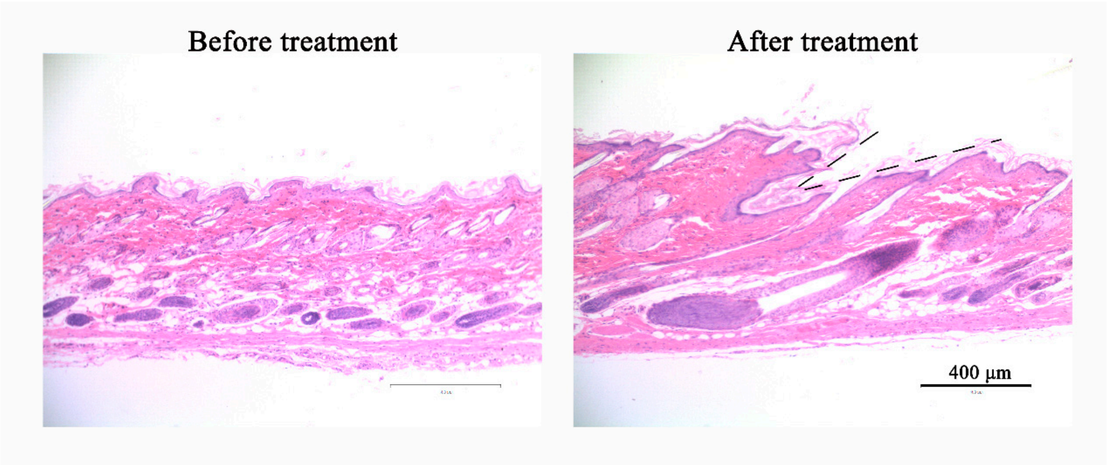

Figure S3. The HE stained sections before and after 808+Lox+Tof NPs@MN treatment.

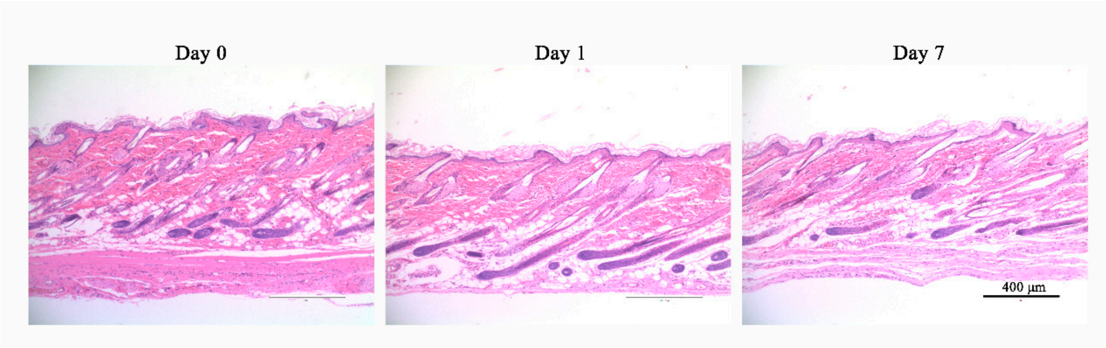

Figure S4. The HE stained sections before 808+Lox+Tof NPs@MN treatment (Day 0) and after 808+Lox+Tof NPs@MN treatment (Day 1 and Day 7).

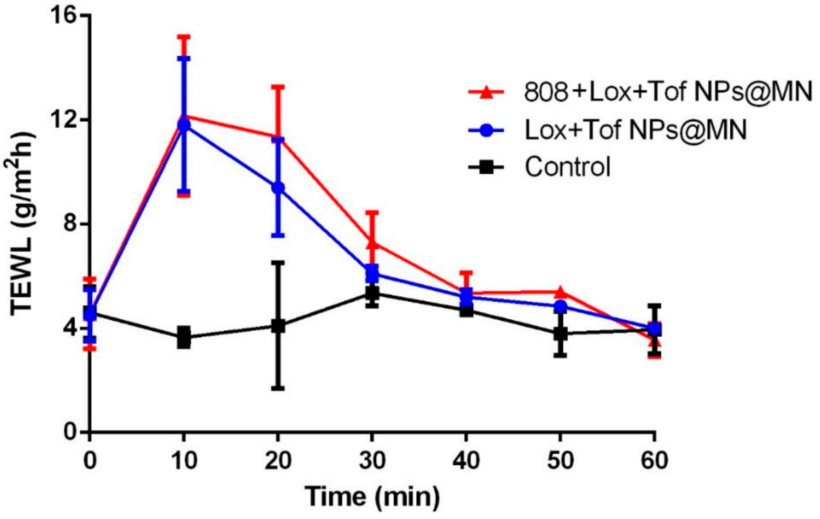

Figure S5. The TEWL after different treatment (Control, Lox+Tof NPs@MN, and 808+Lox+Tof NPs@MN).

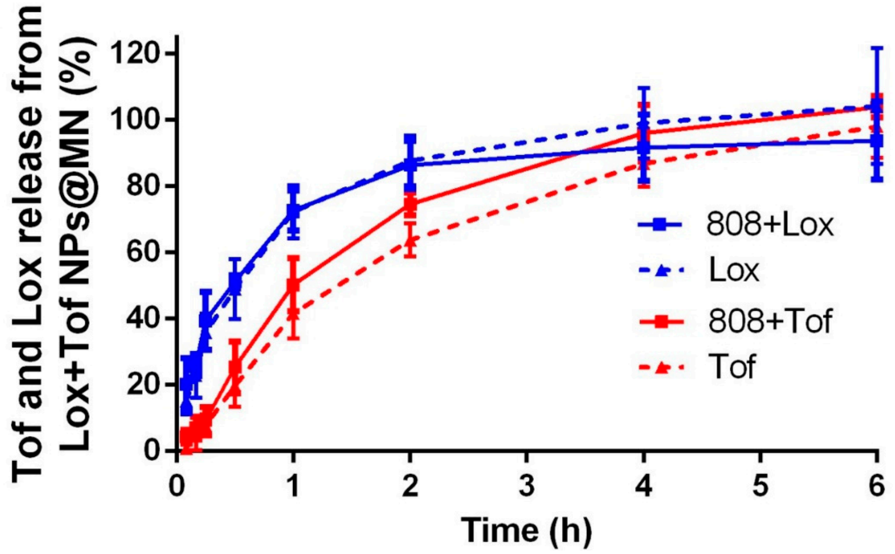

Figure S6. *In vitro* release of Tof and Lox from Lox+Tof NPs@MN. Cumulative release rate of Lox and Tof from Lox+Tof NPs@MNs with or without light irradiation. The irradiation was performed with 808 nm near infra-red light at the power density of 1 W·cm^{-2} for 5min prior to *in vitro* release. (n=4, *$p < 0.05$, **$p < 0.01$, and ***$p < 0.001$).

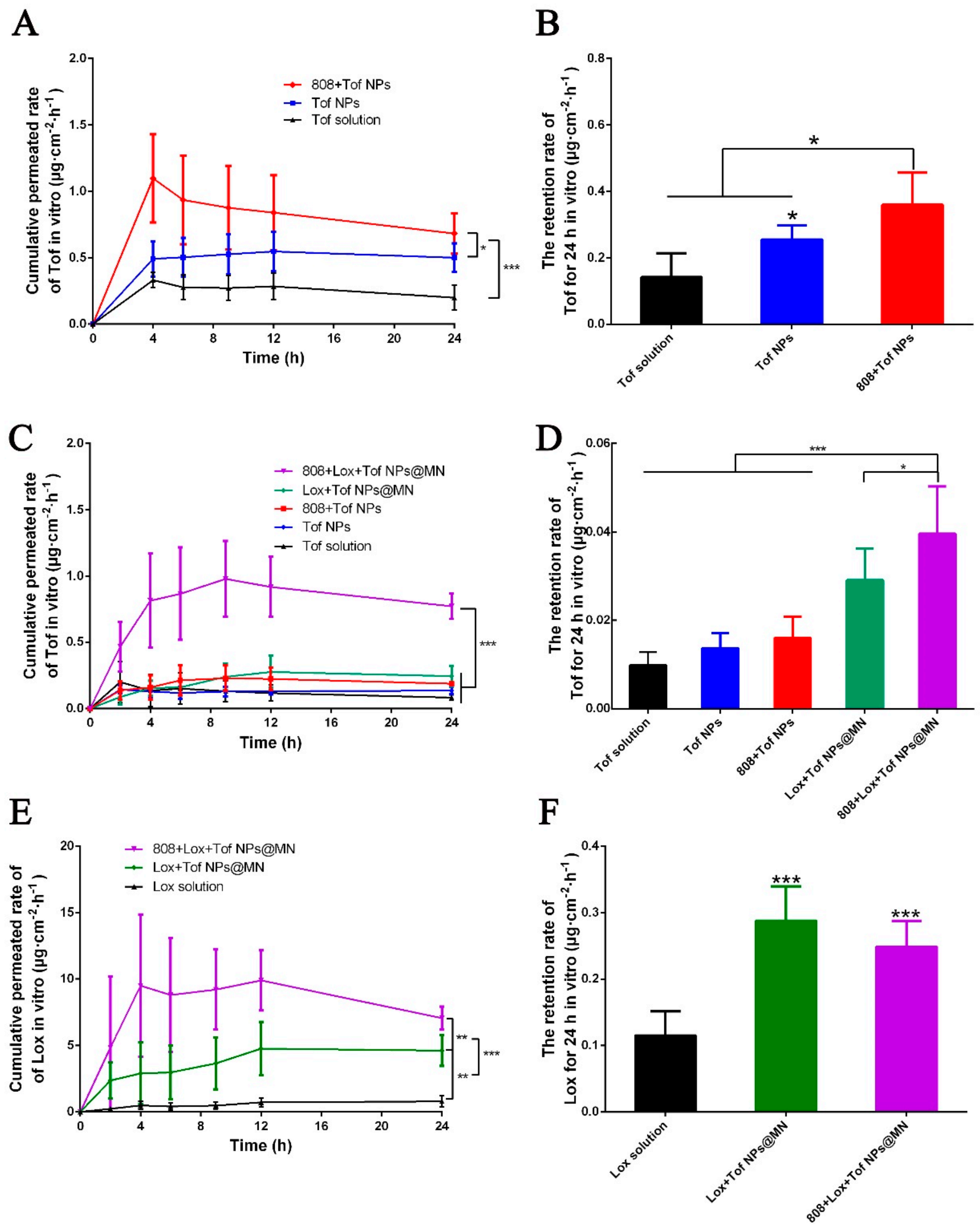

Figure S7. *In vitro* permeation of Tof NPs and Lox+Tof NPs@MN through rat skin for 24h. Cumulative permeation rate (A) and skin retention rate (B) of Tof from Tof NPs with or without light irradiation. Cu-mulative permeation rate (C) and skin retention rate (D) of Tof from Lox+Tof NPs@MN with or without light irradiation. Cumulative permeation rate (E) and skin retention rate (F) of Lox from Lox+Tof NPs@MN with or without light irradiation.

(n=6, *p < 0.05, **p < 0.01, and ***p < 0.001).

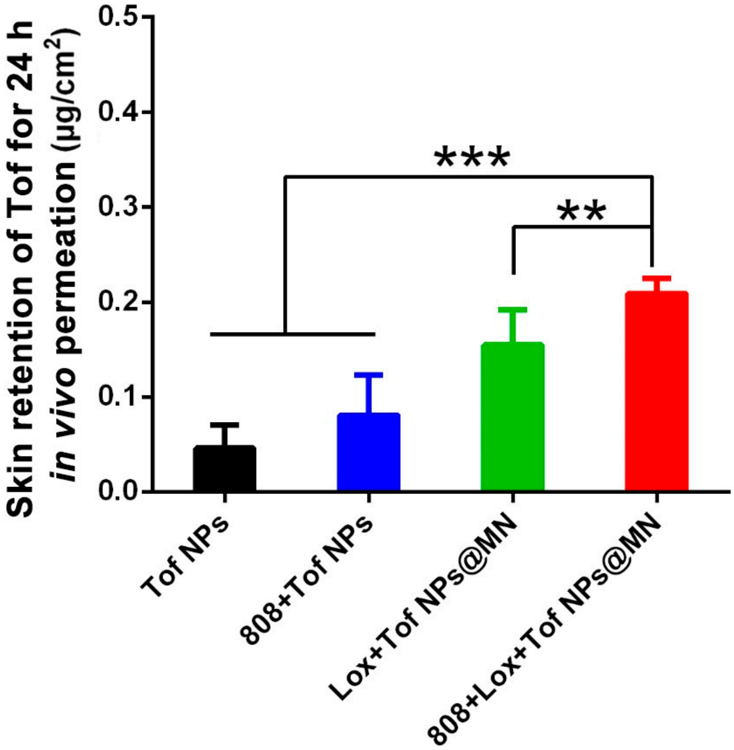

Figure S8. Skin retention of Tof for 24 h *in vivo* permeation from different formulations. (n=4, *p < 0.05, **p < 0.01, and ***p < 0.001)

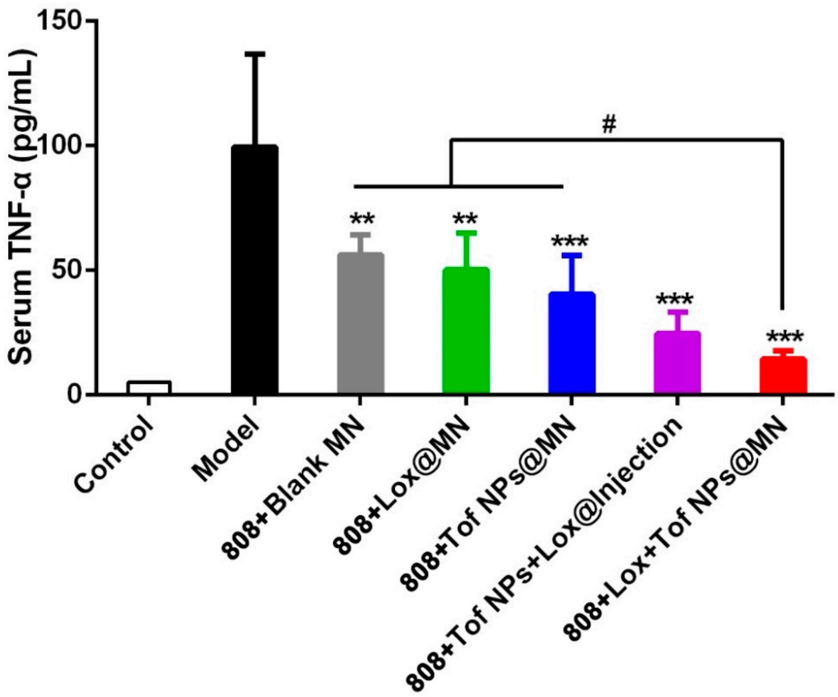

Figure S9. The serum TNF-α of arthritic rats. (n=4,# p < 0.05, *p < 0.05, **p < 0.01, and ***p < 0.001)

Table S1

DNA sequence of primer pairs used for quantitative real time PCR in cells.

Primer	Base sequence (5'-3')
GAPDH (Forward primer)	AGAGGGAAATCGTGCGTGAC
GAPDH (Reverse primer)	CGATAGTGATGACCTGACCGT
iNOS (Forward primer)	CTTGGAGCGAGTTGTGGATTGTC
iNOS (Reverse primer)	TAGGTGAGGGCTTGGCTGAGTG
IL-1β (Forward primer)	ATTGTGGCTGTGGAGAAG
IL-1β (Reverse primer)	AAGATGAAGGAAAAGAAGGTG

Table S2

DNA sequence of primer pairs used for quantitative real time PCR in rats.

Primer	Base sequence (5'-3')
β-actin (Forward primer)	CCCGCGAGTACAACCTTCT
β-actin (Reverse primer)	CGTCATCCATGGCGAACT
STAT3 (Forward primer)	GTAGTGCTGCCCCTTACCTG
STAT3(Reverse primer)	TCCATGTCAAACGTGAGCGA
INOS (Forward primer)	TAGTCAACTACAAGCCCCACG
INOS (Reverse primer)	AGTCACATGCAGCTTGTCCA
TNF-α (Forward primer)	GGCTTTCGGAACTCACTGGA
TNF-α (Reverse primer)	GGGAACAGTCTGGGAAGCTC
JAK2 (Forward primer)	TCCACCCAATCATGTCTTCCAC
JAK2 (Reverse primer)	GCTTCAGCCCCACGAGATAC
JAK3 (Forward primer)	CCTGGAGTGGCACGAGAATC
JAK3 (Reverse primer)	TCCACAACCTCCCGCCTAT
IL-β (Forward primer)	TGCCACCTTTTGACAGTGATG
IL-β (Reverse primer)	AAGGTCCACGGGAAAGACAC
